# Gender and Age Stereotypes in Robotics for Eldercare: Ethical Implications of Stakeholder Perspectives from Technology Development, Industry, and Nursing

**DOI:** 10.1007/s11948-022-00394-1

**Published:** 2022-08-09

**Authors:** Merle Weßel, Niklas Ellerich-Groppe, Frauke Koppelin, Mark Schweda

**Affiliations:** 1 Division of Ethics in Medicine, Carl von Ossietzky University of Oldenburg, Ammerländer Heerstraße 114-118, 26129 Oldenburg, Germany; 2 Section Technology and Health, Jade University of Applied Sciences Oldenburg/Wilhelmshaven/Elsfleth, Ofener Straße 16/19, 26121 Oldenburg, Germany

**Keywords:** Age tech, Care robots, Gender and age stereotypes, Stakeholder perspectives, Qualitative research, Ethics

## Abstract

Social categorizations regarding gender or age have proven to be relevant in human-robot interaction. Their stereotypical application in the development and implementation of robotics in eldercare is even discussed as a strategy to enhance the acceptance, well-being, and quality of life of older people. This raises serious ethical concerns, e.g., regarding autonomy of and discrimination against users. In this paper, we examine how relevant professional stakeholders perceive and evaluate the use of social categorizations and stereotypes regarding gender and age in robotics for eldercare. Based on 16 semi-structured interviews with representatives from technology development, industry, and nursing science as well as practice, we explore the subjects’ awareness, evaluations, and lines of argument regarding the corresponding moral challenges. Six different approaches of dealing with categorizations and stereotypes regarding gender and age in care robotics for older people are identified: negation, functionalistic relativization, explanation, neutralization, stereotyping, and queering. We discuss the ethical implications of these approaches with regard to professional responsibility and draw conclusions for responsible age tech in pluralistic societies.

## Introduction

“[A] perfect Grandma’s (and Grandpa) little helper”, reads a slogan for “Buddy, the emotional robot” (Blue Frog Robotics, 2021). The socially assistive robot is advertised as a promising technological solution to the challenges of population aging and “the future of eldercare” (ibid.). Notably, the shape and user interface of the 56 cm tall device resemble the stature and facial features of a toddler. Indeed, the website lures potential customers with a suggestive question: “How not to resist to his cuteness and not want to adopt him?” (ibid.)

That a robot marketed for use in eldercare is designed to look like and be addressed as a young boy is by no means accidental. Indeed, social-psychological research has established that interaction between humans and robots involves the same social aspects and dynamics as interaction between humans. Users tend to anthropomorphize robots and to assign attributes that influence their attitudes and behavior vis-à-vis their technical counterparts (Eyssel & Kuchenbrandt, 2012). In the field of eldercare, attributions of gender and age traditionally play a particularly prominent and pervasive role in this respect (Sandelowski, 2000).

This social-psychological knowledge is of practical interest for the development and implementation of robots in eldercare. It can elucidate the mechanisms of human-machine interaction in this sensitive area and thus also help to modify it in desirable ways. Some authors explicitly consider using common stereotypes, that is, generalized beliefs about perceived groups like women or older people (McGarty et al., 2002) in order to increase user acceptance, comfort, and quality of care, as illustrated in the introductory example (Jung et al., 2016; Bryant et al., 2020). At the same time, however, such stereotyping strategies give cause for considerable moral concerns. For example, they may undermine user autonomy by subtle manipulation, compromise wellbeing by ignoring individual preferences, and might arguably reinforce prejudice and discrimination, for instance against women or older people (Weßel et al., 2021). Accordingly, there are proposals to design robots without the respective cues and markers, to prevent users from anthropomorphizing them altogether, or even to subvert or challenge stereotypical attributions (Dufour & Ehrwein Nihan, 2016; Eyssel & Hegel, 2012; Wang et al., 2021).

This raises the question of how we can deal with the ambivalent role of gender and age aspects and stereotypes in care robotics for older people in a morally responsible way. Those who are professionally involved in the development and implementation of robots for eldercare in domestic or institutional settings are of particular importance in this context, especially engineers, producers of care robots, and nursing professionals. After all, their perception, understanding, and assessment of the pertinent problems and possible solutions are likely to shape the ways robots will be designed for and utilized in future eldercare. Therefore, we explored the awareness of professional stakeholders from technology development, industry, and nursing science and practice regarding the role of gender and age categorization and stereotypes in robotics for eldercare, their moral significance, and possible solutions or alternatives. In the following, we first provide a brief overview of the state of social-psychological research on stereotypes in robotics and the scope of its morally ambivalent aspects in the context of eldercare. We then explain the methods of data collection and analysis of our own socio-empirical study on stakeholder perspectives. Based on our empirical material, we develop a typology of six different approaches to (stereotypical) social categorizations of gender and age in robotics for eldercare. We discuss the implications of these different approaches from the ethical point of view of professional responsibility and draw conclusions for the responsible development and implementation of age tech in contemporary aging societies.

## Social Categorizations and Stereotypes in (Care) Robotics: State of Research and Ethical Debate

For the development and implementation of robotics, information on the mechanisms of human-technology interaction is of particular interest. Social-psychological research has shown that social categories from human interaction are also effective in the interaction of humans with robots, and work in similar ways in this context. While there are individual studies on aspects of age (Huff et al., 2020; Pak et al., 2020) or race (Addison et al., 2019; Bartneck et al., 2018; Louine et al., 2018; Sparrow, 2020), the bulk of pertinent research concentrates on perceptions of a robot’s gender, the relevant cues and markers, and their effects (e.g., Eyssel & Hegel, 2012; Ladwig & Ferstl, 2018; Nomura, 2017). These cues and markers can be categorized as morphological, vocal, or behavioral, as well as individual-related (Weßel et al., 2021).

*Morphological cues* refer to the robot’s visual appearance. In the context of gender, body shape is one example. Thus, several studies show that waist-to-hip ratio and/or shoulder width can influence the perception of a robot as male or female (Bernotat et al., 2017; Trovato et al., 2018). Facial cues and length of hair also matter (Eyssel & Hegel, 2012). Furthermore, the robot’s voice and *vocal cues* play an important role (Eyssel et al., 2012; Nass et al., 1997). If the voice is clearly identified as male or female, the test person makes assumptions about tasks and competences of the robot that correlate with stereotypical occupations and competences of men and women. For example, a robot with a male voice is perceived as more suitable as a security robot than one with a female voice (Tay et al., 2013). *Behavioural cues* like communication style also have an influence. Thus, studies on stereotypes in verbal human-robot interaction show a strong effect of the implicit gender that manifests, e.g., in stereotypical personality traits (Kraus et al., 2018). The name as one example of an *individual-related cue* also plays a role for the implicit attribution of gender to humanoid social robots (Ladwig & Ferstl, 2018).

Research on the practical effects of such gender cues and markers also makes clear why a stereotypical perception of robots becomes particularly relevant for the development and implementation of robotic technologies in a sensitive field like eldercare. Thus, studies indicate that a robot’s perceived gender also influences the assessment of its features and competences. Especially the assumed suitability for a specific task is closely linked to the perception of its gender (Kuchenbrandt et al., 2014). Other studies show that a ‘male’ robot is evaluated useful for stereotypically male tasks like repairing technical equipment or security activities, while a ‘female’ robot is considered more appropriate for stereotypical female tasks like household and care services (Bernotat et al., 2021). Indeed, Tay et al. (2014) found a slight preference for a female-gendered healthcare robot in contrast to a greater acceptance for a male-gendered security robot. Pointing in a similar direction, a smaller qualitative study revealed that older people applied the idea of nursing as a female task when deciding on a suitable robotic appearance (Rızvanoğlu et al., 2014). Consequently, there are considerations to use stereotypes regarding gender or age in technology development and implementation in order to increase user acceptance and smooth interaction (Bryant et al., 2020; Jung et al., 2016).

Despite these practical effects, there exists no systematic ethical analysis of moral consequences of stereotypes in robotics for eldercare. A first exploration reveals a range of moral aspects regarding autonomy, care, and justice that may affect older people themselves, their caregivers, as well as society at large (for the following, cf. Weßel et al., 2021). In view of *autonomy*, one important question is whether users can make informed and voluntary decisions for or against the implementation of stereotypes. In addition, there is the concern that the continued use of stereotyped robots might lead to a subtle manipulation of user behavior, e.g., by reinforcing or impeding certain communication styles and activities. Eventually, the constant repetition of such effects might also compromise the users’ autonomy by promoting biased perspectives, prejudiced attitudes, and a narrow-minded character. With regard to *care*, stereotypes may have consequences for users’ bodily, psychological and social wellbeing and quality of life. Thus, it is unclear whether the implementation of stereotypes leads to an increase in personal satisfaction, fulfilment, and orientation, or is rather detrimental to users’ wellbeing and flourishing and has negative impacts on the care process. For example, stereotyping may improve comfort and compliance with care robots and thus raise the overall effectiveness of nursing care but can also induce discomfort. Furthermore, stereotyping strategies may compromise a care robot’s regular functioning and cause malfunction, misoperation, or safety risks. Eventually, long-term influences on the users’ fundamental preference structure must be taken into consideration, for example an encouragement of sexist attitudes or even transgressive behavior. With regard to *justice*, a central question is whether users have equal access to stereotypical and non-stereotypical robots and their respective benefits or disadvantages. A further question is how the implementation of stereotypes affects users’ possibilities of participation and inclusion, e.g., with regard to highly gendered areas of social life. Finally, the very idea of stereotyping may contradict fundamental principles of dignity and justice that call for equal respect and mutual recognition of all individuals. Although pertinent empirical evidence is still scarce and inconclusive, there are concerns that stereotyping strategies could affirm and reinforce existing stereotypes and societal bias, injustice, and discrimination.

So far, there is hardly any systematic empirical research on public views of these moral issues. Especially their perception and evaluation among those professionally involved in the development and implementation of robots for eldercare, e.g., representatives from engineering and design, robotics industry and nursing, is largely unknown. Yet, the perspectives of these professional stakeholders are highly relevant in this context as they will influence how robots for eldercare will be developed and used in the future. This key role and societal influence come with considerable responsibility: Professional stakeholders’ decisions regarding construction, design, marketing, and practical utilization of robots for eldercare can promote or undermine the intended effects due to unforeseen influences of stereotypes on functionality or user acceptance. They may even reinforce (or alleviate) serious moral problems concerning manipulation, wellbeing, or societal discrimination. Therefore, it is crucial to investigate to what extent professionals from technology development, industry, and nursing care are aware of the role of (stereotypical) categorizations regarding gender and age in robotics for eldercare, its moral significance, and possible solutions or alternatives. After all, this moral awareness of possible implications and consequences of their own work constitutes a necessary precondition for the assumption of professional responsibility and thus for the responsible development of age tech.

## Methodology

To explore the views of professional stakeholders on social categorizations and stereotypes in robotic eldercare and their awareness of the pertinent moral issues, we chose an explorative-qualitative approach. We conducted 16 semi-structured interviews with professionals from technology development, marketing, as well as nursing science and practice. The study protocol (Nr. 2021-41) was approved by the Research Ethics Committee of the School for Medicine and Health Sciences at the University of Oldenburg. A review of the project was also conducted by the data protection officers of the participating universities.

The four professional domains were selected to represent the relevant areas of developing care robotics (technology development), devising strategies for their commercial distribution (marketing), evaluating their implementation (nursing science) and practically utilizing robotic technologies (nursing). The inclusion criterion for all four domains was that potential interviewees were knowledgeable and experienced in their field regarding robotics for eldercare. The interviewees in the nursing group either had a background in professional nursing or were involved in the implementation of robotics in a care institution. All of them were either employed in formal outpatient care or in a long-term care facility. Further inclusion criteria were a minimum age of 18 years and sufficient German language proficiency. Recruitment was based on comprehensive online research and existing networks. Potential interview partners identified were approached personally. Snowball sampling was also used to recruit further participants who met the inclusion criteria. The aim was to include an equal number of professional stakeholders from each area. Table 1 shows the final sample by gender and professional domain.

**Table 1:** Sample description by gender and professional domain (N = 16)

Domain	Female	Male
Technology development	1	3
Marketing	1	3
Nursing science	1	1
*Nursing (nursing facilities, formal outpatient care, long-term care, welfare associations, services)*		
Management level	1	2
Implementation level	1	1
Practice level	1	-
Total	6	10

The semi-structured interview guideline focused on the stakeholders’ perceptions of the users (especially in relation to age and gender) and their presumed needs and preferences. In addition, we addressed the significance and consideration of these needs and wishes as well as the relevance of age and gender stereotypes in the development and implementation of care robotics. Another focus of the guideline was on the perception and evaluation of ethical aspects of stereotyping in human-technology interaction. For this purpose, the strategic use of stereotypes was explicitly addressed in the interview guide and a critical discussion of such strategies was stimulated.

Data collection took place online between April and June 2021 via video conference with the help of the BigBlueButton conference system (BigBlueButton Project, 2020). The interviews were audio-recorded with a digital recording device. The video material was not recorded. Socio-demographic data (name, age, contact details, profession, affiliation) as well as information on professional expertise were also collected prior to the interviews to characterize the sample and to have further contextual information for the analysis. The recordings of the interviews were transcribed verbatim and anonymized. A computer-assisted content analysis using MAXQDA analysis software (VERBI, 2020) was carried out by two of the authors of this paper (Kuckartz, 2018). The data was first coded deductively with a set of codes to understand the interviewees’ evaluation of the relevance of gender and age as well as their moral evaluation. In a second step, the data was coded inductively creating further codes for aspects emerging from the material, for example further relevant social aspects beyond age and gender. To ensure intercoder reliability, two researchers coded the same document separately and the coding system was revised accordingly. During the coding process, the researchers worked closely together and discussed possible problems on a regular basis.

## “Our Robot is Perceived as Very Female, for Whatever Reason” – Professional Stakeholder Perceptions of Social Categorizations and Stereotypes in Robotics for Eldercare

In our interviews with professional stakeholders, various aspects of age and especially gender in care robotics were discussed. In the systematic analysis of the data material, we could distinguish six different types of approaches to the role of the respective social categorizations and stereotypes in this context. Each involves different views and evaluations regarding technology, human-robot interaction, and social stereotypes in the context of robotics in eldercare.

The first approach can be classified as *negation*. In this perspective, no significance is ascribed to social attributions and their relevance is denied or ignored. For example, one interviewee with a background as a care worker stated: “I believe in the end it does not matter, does it? (…) These gender roles, (…), I mean, they do not play a role” (I12, 59). Frequently, this assessment was accompanied by a scientific-mechanistic understanding of technology that reduced the robot to its seemingly objective causal mechanisms and framed social attributions as mere subjective projections that cannot be explained in a rational way and thus should not be taken seriously in the context of robotics. Thus, another interviewee from the field of engineering explained: “As an engineer, you can’t expect that we look at a robot and project something into it like an ordinary person. To me, a robot is a machine. (…) It has no gender in that sense. It is a mechanical construction that is subject to specific physical-mechanical conditions” (I1, 53). One technology developer stated that “at no point there was a need that we differentiate between male and female care workers” (I14, 80) during the development and implementation of care robots.

By contrast, representatives of the *functionalistic relativization* approach admitted the existence of social categorizations in robotics but considered them negligible vis-à-vis the functionality of the robot. For example, one technology developer declared: “I think especially in care, functionality should be above all” (I13, 63). In this context, the function was frequently defined in terms of the care process and its practical requirements and outcomes. Thus, another person from technology development stated with regard to a care robot: “(…) for us, it is important, as I said, that it is really useful and that it fulfills its tasks, and that is more important than these perceptions of roles” (I9, 43). According to an engineer, the main interest was to “solve a problem” (I1, 93). Interestingly, emotional qualities were frequently defined as essential for a robot’s functioning in eldercare but were at the same time deemed independent from social ascriptions. For example, one researcher in nursing studies explained that “for me personally it would hardly matter if the robotic system speaks with a male voice or not. The voice must inspire trust (…)” (I2, 69). A care worker and manager of a care home emphasized that the voice must be “empathic” and “affectionate” (I3, 33). Overall, this approach was especially prominent in interviews with professional caregivers, professionals in care homes, and nursing scientists.

In contrast to the first two types, the approach of *explanation* assigned more significance to social aspects. Its representatives acknowledged the relevance of social categorizations and stereotypes in human-robot interaction but suggested to avoid them by explaining the functionality of the robot as a machine. An interviewed researcher said: “Hence technology development is very deliberate and strategic. And look at these new Fraunhofer-things they put on the market. They are not humanoid anymore, that is not a care robot anymore, that is really deliberately and strategically a technical device. (…) There is a strategy behind that” (I6, 27). Accordingly, many representatives of this type argued that a reference to stereotypes could be prevented by explaining the functionality and technological characteristics to the users. For example, one interviewed person stated that ideas of gender and age could be observed especially when the robot was introduced and the users had no idea or no experience with robots (I9, 23). According to her, the initial explanation of a robot’s functionality could help to defuse possible attributions of social categories since the users are made to realize that they are dealing with a machine and not a social agent. In this perspective, social categories and stereotypes were primarily seen as an indicator for a lack of technology competence and experience so that the increase of this competence would decrease the attribution of social categories to robots.

Another attempt at defusing social categorizations and stereotypes was based on the approach of *neutralization*. Its representatives acknowledged the relevance as well as sometimes the problematic aspects of social categories and stereotypes in human-robot interaction and therefore suggested to create care robots that do not have any social markers like gender cues. For example, the look should neither be female or male, the voice should be gender neutral and the robot should act in a gender-neutral manner. An engineer explained: “So, female, male or there are also these neutral voices in between, the ones that do not permit any gender attributions at all. That’s something one can do, but it is more difficult from a technical point of view” (I1,83). According to its proponents, this approach could help to circumvent morally contested issues like the reproduction of gender stereotypes in care “because you can probably only get into trouble with that [i.e. gender attributions]” (I16, 41). One marketing expert argued that a neutral approach was more inclusive and facilitated the establishment of a personal relationship: “At the moment everything is gender neutral. Pepper addresses people mostly with ‘you’. That certainly is a door opener because you do not address a person as ‘mister’ or ‘miss’ but directly create a personal connection” (I4, 27). A care worker also suggested that a care robot should be gender neutral: “And I think it is somehow important, that (…) when a robot is used (…), it should be kept more neutral” (I12, 63). This interviewee suggested further that “maybe this helps to clarify that nursing and so on is not a female job (…)” (I12, 65). Another interviewee who is responsible for implementing technology in a care home also stated: “I would keep it without gender” (I11, 79–80).

Two further types acknowledged the efficacy of social categorizations in care robotics and proposed to integrate them in technology development and implementation. A first example for such an integrative strategy is *stereotyping*. In this case, the strategical use of social categories and stereotypes was seen as an option to increase acceptance and compliance in human-robot interaction. For example, a person from marketing stated: “We deliberately went for the childlike track. We were very, very purposefully on the `Hello Kitty’ track concerning appearance and design. […] I would even argue that the childlike, the cuteness is very important for the robot’s design” (I7, 55). Another marketing expert explained the rationale behind such a strategy: “(…) because you are actually always nice to children, you are very open, you try to support them, or you try to interact with them. And that is what Pepper has achieved“ (I4, 57). Other participants would also be open to such strategies as long as their effectivity was scientifically validated. Thus, a technology developer stated: “When research shows that we can increase the acceptance if we give it a female name or so. Or adapt the looks accordingly. Why not, I would say (…)” (I16, 29). The aforementioned marketing expert said: “Yes, if it is helpful. If it improves the behavior towards robotics and increases the acceptance. Yes, of course. Then one can do that” (I4, 53). Another technology developer was convinced that “(…) in case it leads to a greater acceptance of the technology, so that anxiety [i.e. having a robot] decreases, then it is absolutely okay” (I13, 59).

Finally, the concept of *queering* turned out to be fruitful to subsume approaches that explicitly considered social and political dimensions of stereotypes in care robots. The respective interviewees were aware of the ambivalent consequences of stereotypes and possible discriminatory effects. According to them, discrimination must be avoided, and the preferences of non-heteronormative people respected in robotics. A technology developer reflected: “Yes, generally, the problem with perceptions of roles is that if possible no stereotypes are implemented (…) this might result in some people rejecting it (i.e. the robot) or feel discriminated because they feel this way about the subject. For this reason, I think, the system must be flexible enough and be able to adjust individually” (I16, 39). This fits well with the critical perspectives we found in our interviews with regard to stereotypes. For example, one scholar of nursing studies stated: “I struggle with the idea to go on developing these gender stereotypes just like that. I am not sure, whether this is so good” (I2, 69). Especially regarding gender stereotypes, several interviewed persons referred to the “societal problem” (I11, 67), to reproduce and reinforce stereotypes and clichés in technology development (ibid.; I14, 109 + 115; I1, 77). Furthermore, not only individual preferences were important in this *queering* approach but a particular focus was placed on people and preferences that might contradict common social norms. An interviewed person leading a project to implement care robots in care homes said: “And the robots should be designed as colorful as humans are” (I11, 67). The aim is a robot that acknowledges the importance of social categories in human-robot interaction but goes beyond their stereotypical implementation. This strategy focused on the individuality and diversity of the users and attributed importance to this individuality: “So instead of classifying patients, which is always difficult, I have to consider the individual needs, possibilities and demands of the person” (I1, 71). Hence, despite the actual technological limitations, the need for individual configurations and customization of the technology was emphasized (I16, 47; I1, 75). Especially with technological progress, the opportunity for even more flexible technology was seen. For example, a person from technology marketing stated: “In the long run, digital services will always be freely selectable. It is a little bit such an avatar-system, where we see: Yes, alright, each according to their own” (I15, 20). The possibility that this personalized approach could in fact reproduce existing user stereotypes was not discussed.

## Towards a Responsible Approach to Social Aspects in Age Tech

The development and implementation of technology in the sensitive area of eldercare calls for a particular sense of responsibility on the part of those involved. It requires at least some awareness of the relevant facts and (psychosocial) mechanisms as well as suitable normative standards in order to detect and tackle potential moral problems (Schicktanz & Schweda, 2012). This also comprises the effects of social categorizations and stereotypes in care robots for older people.

In our interview study with stakeholders, we could extract six different approaches to such categorizations and stereotypes. They represent ideal types that frequently overlapped and blended in the actual interviews. Some of them have already been discussed in previous research, for example, ideas of explanation (Dufour & Ehrwein Nihan, 2016), neutralization (Eyssel & Hegel, 2012), stereotyping, or queering (Wang et al., 2021). Each of these approaches involves different potentials and problems for the responsible development and implementation of robots for eldercare.

Representatives of the *negation* approach show little awareness of the relevance and potential problems of social aspects in care robotics. This lack of awareness makes it hard to devise responsible ways of dealing with social categorizations and stereotypes. By neglecting the socio-cultural dimension of human-robot interaction, the negation-approach runs the risk of promoting a thoughtless and therefore reckless use of social categories. At the same time, the beneficial potentials of social attribution and even stereotyping in robotics are also not taken into consideration. Altogether, this approach can unwittingly and unwillingly cause and reinforce moral problems, for example discriminatory consequences of implicitly implemented stereotypes that might affect users’ wellbeing or social standing.

The approach of *functionalistic relativization* makes clear that there are also good technological reasons for taking social aspects of technology into account. Although the approach shows some awareness of social categorizations, it focuses on functionality alone and therefore draws no practical consequences. Such an approach also tends to neglect possible effects of social categorizations and stereotypes in robotics on functionality, be they beneficial or detrimental. For example, the potential of strategic uses of stereotypes to improve acceptance and compliance and thus to increase the wellbeing of users is not considered. Hence, the proponents’ objective of achieving optimal functionality of care robots is undermined by their own neglect of the relevance of social aspects for good functionality.

Other approaches can at least be understood as attempts to tackle these social aspects, albeit the means may fall short in the end. Thus, the strategy of *explanation* comprises a certain awareness regarding social categorizations, stereotypes, and their problematic implications. At the same time, however, these social attributions are only considered as the result of a lack of expertise and technological know-how on the part of the users (Dufour & Ehrwein Nihan, 2016). Following this information-deficit-logic, the approach is aimed at avoiding social attributions by giving explanations about technical characteristics. This expertocratic perspective systematically underestimates the significance and effectivity of social aspects in human-robot interaction. It therefore also neglects the potentially helpful and beneficial consequences of stereotypes, such as increases in user wellbeing or acceptance.

The approach of *neutralization* also shows some awareness for social categories and their problems but ultimately relies on a purely technical fix to avoid them. It is aimed to eliminate social categorizations and their implications for human-robot interaction by means of neutral technology development and design. However, this attempt also underestimates the pervasiveness of social aspects in human-robot interaction. The idea of a truly neutral technology is an illusion. Even a robot that was perceived as neutral by the developer might not be experienced as neutral by the users. For example, although the service robot Pepper was intended to be gender-neutral, users nevertheless tend to assign a gender (Bryant et al., 2020). Like the aforementioned approach, the idea of neutralization also neglects that social categories are an inevitable aspect of any social interaction. Even if it was possible to create a truly neutral robot, it might therefore raise discomfort and distrust and irritate users. Therefore, the attempt to circumvent the social aspects of human-robot interaction proves to be insufficient for addressing the relevant ethical challenges in a responsible way.

The last two approaches show most awareness for social aspects in robotics and promote their productive integration in technology development. Yet, in doing so, they go in opposite directions. The *stereotyping*-approach focuses on the beneficial effects of using stereotypical attributions and categorizations and neglects the detrimental consequences. This approach favors a deliberate use of stereotypes to increase user acceptance and comfort. On the one hand, this can be considered as an approach to increase the wellbeing and quality of life of users by using stereotypes to create a bond between robot and user. On the other hand, however, it might amount to a manipulation of users. Stereotypes might be used to lure reluctant users into acceptance of a robot against their own preferences. This might compromise the autonomy and self-determination of the user. With its focus on acceptance and compliance, this approach also neglects the diversity of the users as well as possible broader social and political consequences of social categorization and stereotyping. This may run the risk of reproducing discrimination and injustices by reinforcing common stereotypes.

By contrast, *queering* approaches recognize the societal and political implications of social attributions in human-robot interaction, for example the further marginalization or even discrimination of already marginalized groups. Its proponents favor robots that are as heterogenous as humans. In this sense, they challenge oversimplified applications of social categories since they could harm or neglect non-heteronormative users and undermine social diversity. However, the normative implications of the idea that robotics should help every individual to develop and realize their own identity need further elaboration. On the one hand, a merely personalized approach tailored to individual user preferences might in fact simply reproduce existing stereotypes. On the other, queered robots might also have detrimental effects on people with heteronormative orientations. The question is to what extent there is a moral responsibility of robotics to challenge common stereotypes and indeed make people uncomfortable in order to stimulate social change. Depending on the normative weight placed on such a subversive approach, it may be justified to subordinate individual autonomy or wellbeing for the sake of justice for non-heteronormative and marginalized groups (Ellerich-Groppe et al., 2021).

In the face of the ambivalent role of social categorizations and stereotypes in human-robot interaction, our research highlights the perspectives of stakeholders from technology development, marketing, and nursing science and practice. Our small qualitative analysis was aimed at a first exploration of their views and attitudes. Similar to the academic debate, the significance of gender was discussed extensively in the interviews while aspects of age did not receive comparable attention and therefore still require further examination. In addition, further research is needed to include the perspectives of other user and stakeholder groups, especially caregivers and cared-for people. Since the study took place in the German-speaking area, research focusing on the international development would also be important to complement our study and highlight national similarities and differences. Finally, larger quantitative approaches would be necessary to generate representative results.

Nevertheless, a number of important conclusions can be drawn from our results. Since the use of social attributions in the context of robotic care can have serious and morally problematic implications, it raises questions of professional responsibility that require an empirically informed ethical analysis. In general, the ascription and acceptance of responsibility implies awareness of the relevant facts and normative standards to detect and tackle potential problems. On this basis, relevant preconditions and obstacles for responsible technology development and implementation in eldercare can be identified regarding the six approaches identified in our study. In fact, each of these approaches involves different degrees of awareness as well as different normative premises. Especially the first two types – *negation* and *functionalistic relativization –* lack awareness of the relevant mechanisms of human-robot interaction and of its potentially morally problematic aspects. Instead, their proponents seem to see their responsibility in other areas, for instance the technological functioning of care robotics. The third and fourth type – *explanation* and *neutralization* – could at least benefit from a more socio-culturally informed and reflected understanding and handling of social aspects in care robotics. The last two types – *stereotyping* and *queering* – explicitly acknowledge the role of social categorizations but need further discussion regarding the normative standards underlying their – either affirmative or subversive– attitude vis-à-vis stereotypes in robotic eldercare. Especially the one-sided focus of stereotyping-strategies on individual wellbeing and acceptance, as well as the concrete normative orientation of queering approaches, appear to be worth discussing.

On the whole, these remarks underline the need for more interdisciplinary approaches in technology development and implementation that consider ethical and social scientific findings at every stage. Interdisciplinarity should already be considered in robotics education that needs to integrate social scientific and ethical knowledge and competence regarding human-robot interaction. The future of age tech not least depends on the question to what extent ethics, social sciences, and technology development will be able to cooperate and integrate each other’s perspectives. If they succeed, this can provide the ground for responsible, diversity-sensitive technologies for eldercare in a pluralistic society.
